# Case Report: From intellectual disability to fatal cytokine storm: long-term follow-up reveals a novel systemic phenotype in METTL23-related disorder

**DOI:** 10.3389/fped.2026.1991266

**Published:** 2026-10-26

**Authors:** Yishan Zhan, Jian Zha, Hui Chen, Yuanyuan Chen

**Affiliations:** 1 Department of Pediatric Intensive Care Unit, Jiangxi Provincial Children’s Hospital, Nanchang, China; 2 Department of Neurology, Jiangxi Provincial Children’s Hospital, Nanchang, China

**Keywords:** autoimmune hemolytic anemia, case report, cytokine storm, hemophagocytic lymphohistiocytosis, intellectual disability, METTL23

## Abstract

METTL23 is a rare gene encoding a lysine/arginine methyltransferase associated with autosomal recessive intellectual disability 44 (MIM 615942). To date, published cases have been limited to neurodevelopmental phenotypes, with recent mechanistic work also implicating METTL23 in histone H3R17 methylation and negative regulation of NF-*κ*B–mediated proinflammatory signaling. We previously reported the neurodevelopmental phenotype of a Chinese girl with a novel homozygous METTL23 splice variant (c.322 + 1del). Here we report the long-term follow-up and fatal outcome of the same patient. Over a 3-year follow-up period, the patient experienced three episodes of autoimmune hemolytic anemia and a secundum atrial septal defect treated by percutaneous closure. At 4 years and 6 months, she was admitted with fever and cyanosis progressing to Epstein–Barr virus-associated cytokine storm with features overlapping, but not fulfilling, HLH-2004 criteria; the episode was managed clinically as septic multi-organ dysfunction. The illness was complicated by acute kidney injury requiring three sessions of continuous renal replacement therapy, myocarditis, and methicillin-resistant Staphylococcus aureus superinfection. Despite intensive multidisciplinary treatment, the illness progressed to multifocal intracranial hemorrhage with herniation, and the family elected to discontinue care; the patient died shortly thereafter. This case extends the phenotypic spectrum of METTL23-related disorder beyond neurodevelopmental impairment to include recurrent autoimmune cytopenia and a fatal virus-triggered cytokine storm. Given the reported role of METTL23 in restraining NF-*κ*B–driven cytokine production, we hypothesize that loss-of-function variants may confer susceptibility to dysregulated inflammatory responses upon viral triggers, independent of whether formal HLH criteria are met. Long-term multidisciplinary surveillance, including vigilance for infection-triggered hyperinflammation, may be warranted in patients with METTL23-related disorder.

## 1 Introduction

*METTL23* (methyltransferase-like 23) encodes a small, 190-amino-acid lysine/arginine methyltransferase first identified in 2014 as a novel cause of autosomal recessive intellectual disability (ID) in two independent consanguineous pedigrees of Arab and European/Pakistani origin (1, 2). METTL23 physically interacts with the transcription factor GABPA and is required for normal transcriptional activity at GABP target genes such as *THPO* and *ATP5B* (1). Subsequent reports from Saudi Arabia, Pakistan, Morocco, and China have expanded the mutational spectrum of *METTL23*-related ID (OMIM #615942), consistently describing global developmental delay, mild-to-moderate cognitive impairment, and variable facial dysmorphism, with corpus callosum thinning and delayed white matter myelination as recurrent neuroimaging findings; behavioral abnormalities (including attention-deficit/hyperactivity disorder and aggressive behavior), seizures, and additional neuroimaging findings such as hippocampal asymmetry and ventriculomegaly have also been described in a subset of patients (3–6).

Beyond the nervous system, emerging evidence suggests that *METTL23* may have broader, tissue-specific functions. A three-generation family study identified *METTL23* as a novel gene for normal-tension glaucoma, demonstrating that METTL23 catalyzes dimethylation of histone H3R17 and is required for transcription of *pS2*, an estrogen receptor *α* target gene that negatively regulates NF-*κ*B–mediated TNF-α and IL-1β feedback in retinal ganglion cells (7); this gene-disease association was independently replicated in a separate cohort (8). More recently, a case report described a boy with *METTL23*-related developmental delay who developed intravenous immunoglobulin-resistant Kawasaki disease with giant coronary artery aneurysms, a cytokine-driven vasculitic disorder of childhood (9). Taken together, these observations raise the possibility that loss of METTL23 function may predispose to dysregulated NF-*κ*B–dependent inflammatory responses, in addition to its established role in neurodevelopment.

Hemophagocytic lymphohistiocytosis (HLH) is a life-threatening hyperinflammatory syndrome driven by excessive cytokine release, diagnosed according to the HLH-2004 criteria when five of eight features are present: fever, splenomegaly, bicytopenia, hypertriglyceridemia and/or hypofibrinogenemia, hemophagocytosis, hyperferritinemia, elevated soluble interleukin-2 receptor, and low/absent NK-cell activity (10). In children, secondary HLH is most commonly triggered by Epstein–Barr virus (EBV) infection. Not all EBV-triggered hyperinflammatory episodes meet full diagnostic criteria, however, and may instead be classified as EBV-associated cytokine storm or systemic inflammatory response, particularly when splenomegaly and marrow hemophagocytosis are absent. To our knowledge, no prior report has described either HLH or a sub-threshold hyperinflammatory/cytokine storm presentation in a patient with a *METTL23* variant.

Here, we report the long-term follow-up of a girl with a homozygous *METTL23* splice variant (c.322 + 1del), whose neurodevelopmental phenotype was previously described by our group (6). Over the subsequent three years, she experienced recurrent autoimmune hemolytic anemia and, ultimately, a fatal EBV-triggered episode of systemic hyperinflammation. Although several laboratory features overlapped with HLH-2004 criteria, the absence of splenomegaly and marrow hemophagocytosis did not support a formal HLH diagnosis, and the episode was managed clinically as EBV-associated cytokine storm with septic multi-organ dysfunction. This case extends the phenotypic spectrum of *METTL23*-related disorder and raises a novel hypothesis regarding its potential role in immune regulation.

## 2 Case presentation

### 2.1 Patient background and genetic diagnosis

The patient, a girl born on December 30, 2021, was able to lift her head at 4 months, roll over at 5 months, and sit unassisted at 10 months, but presented with developmental delay noted at 1 year 3 months of age, characterized by inability to crawl, a preference for reclining, inability to stand with support, and poor hand dexterity, accompanied by generalized hypotonia. Family history was notable for retinitis pigmentosa in the father. Trio-based clinical exome sequencing (iWES) performed at 1 year and 3 month of age identified a homozygous splice-site variant in *METTL23* (NM_001080510.5: c.322 + 1del), inherited from a heterozygous carrier father; this variant was classified as pathogenic per ACMG guidelines (PVS1 + PM3_Supporting + PM2_Supporting) and was not present in gnomAD population databases. Five additional variants of uncertain significance were identified in *TTC8, LAMA2, PC, CPS1*, and *PPP1R21* but were not considered to explain the patient's phenotype. This patient's neurodevelopmental phenotype, genetic findings, and neuroimaging features were previously reported by our group (6).

### 2.2 Interval clinical course (may 2024–January 2026)

Over the following three years, the patient was hospitalized on five additional occasions prior to her final admission (Figure 1). At 2 years 4 months, she presented with fever, pallor, and a single seizure-like episode. She had severe anemia (hemoglobin 76 g/L) with a positive direct antiglobulin test, consistent with autoimmune hemolytic anemia (AIHA). Bone marrow examination showed reactive erythroid hyperplasia without malignancy, and echocardiography incidentally revealed a secundum atrial septal defect (ASD). She responded to methylprednisolone. AIHA recurred at 3 years 3 months, with concurrent bronchopneumonia, and again at 3 years 9 months, with Coxsackievirus A16 hand-foot-mouth disease. Both recurrences were triggered by infection and responded to corticosteroids. At 3 years 9 months, EEG showed epileptiform discharges over the central and centrotemporal regions. Video-EEG monitoring at 4 years 0 months led to a revised diagnosis of gratification disorder rather than epilepsy, although background abnormalities persisted. The ASD was closed by transcatheter occlusion without complications at 3 years 11 months.

**Figure 1:**
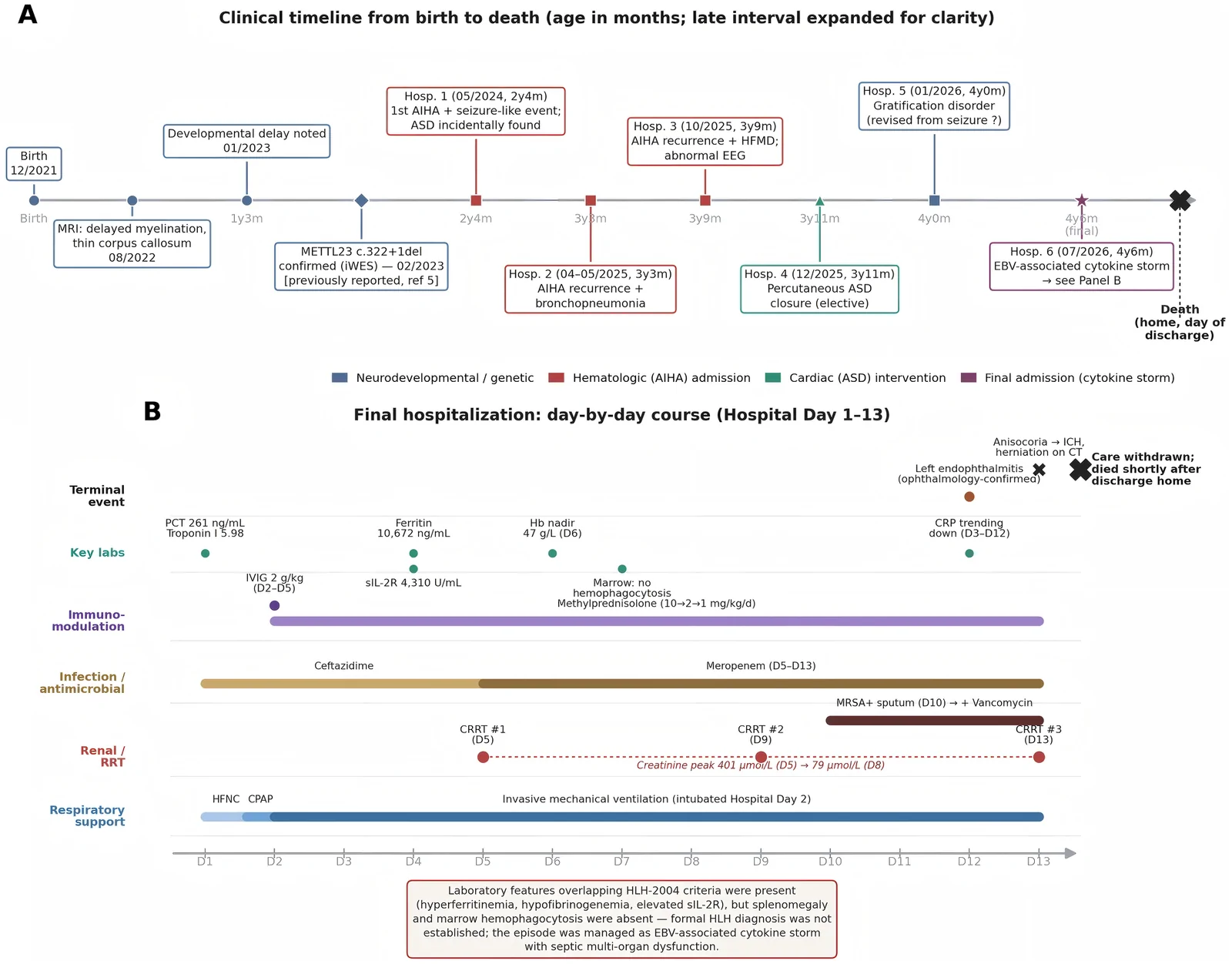
Clinical timeline of the patient. The upper panel shows key neurodevelopmental, hematologic, and cardiac events and prior hospitalizations from birth to the final admission. The lower panel details the final hospitalization by hospital day, including key laboratory results, immunomodulatory and anti-infective treatments, renal replacement therapy, respiratory support, and terminal events. Ages are shown at each event. AIHA, autoimmune hemolytic anemia; ASD, atrial septal defect; CRRT, continuous renal replacement therapy; EBV, Epstein-Barr virus; MRSA, methicillin-resistant Staphylococcus aureus.

### 2.3 Final hospitalization: EBV-associated cytokine storm

At 4 years 6 months of age, the patient presented with a 2-day history of fever (up to 40.0 °C) and cough, treated at a local clinic with antipyretics, cefixime, and ribavirin without improvement. On the day of admission, she developed tea-colored urine, abdominal pain, vomiting, and perioral cyanosis. On admission, she was tachycardic (120/min), tachypneic (30/min), and hypoxic with cyanotic facies; blood pressure was 105/58 mmHg. Initial investigations showed leukocytosis (17.15 × 10⁹/L), anemia (hemoglobin 112 g/L), markedly elevated transaminases (AST 517 U/L), hyperbilirubinemia, and elevated procalcitonin (261 ng/mL).

Over hospital days 2–3, she developed progressive respiratory failure requiring endotracheal intubation with mechanical ventilation. Echocardiography showed a small pericardial effusion and mildly reduced left ventricular systolic function without residual ASD shunt. Laboratory findings indicated a multi-system hyperinflammatory process: coagulopathy (INR 2.2, fibrinogen 0.76 g/L, D-dimer 60 mg/L), markedly elevated cardiac (troponin I 5.98 ng/mL) and hepatic (AST 778 U/L) enzymes, and acute kidney injury (creatinine rising to 267 μmol/L) with oliguria.

Epstein–Barr virus DNA was detected in throat swab (2.47 × 10^3^ copies/mL) and, subsequently, in blood by metagenomic next-generation sequencing. Serial EBV serology was available. At 1 year 10 months of age, VCA-IgG, VCA-IgM, EBNA-IgG, and EBV DNA were all negative. At 2 years 4 months, VCA-IgG and EBNA-IgG were positive and VCA-IgM was negative, indicating an interval asymptomatic primary infection, and this pattern persisted at subsequent admissions. During the final admission, EBNA-IgG remained positive, VCA-IgM was negative, and VCA-IgG had become negative. This pattern is consistent with EBV reactivation rather than primary infection. The loss of VCA-IgG occurred without prior plasma exchange or IVIG and may reflect impaired humoral immunity.

By hospital day 4, serum ferritin (10,672 ng/mL) and soluble IL-2 receptor (CD25) was elevated (4,310 U/mL). However, splenomegaly never developed, and bone marrow examination on hospital day 7 showed reactive erythroid hyperplasia without hemophagocytosis. NK-cell percentage was low (2.71% of lymphocytes; functional activity not assessed) and complement C3, C4, and C1q were all reduced. Given the absence of splenomegaly and marrow hemophagocytosis, the treating team did not establish a formal diagnosis of HLH; The presentation was classified as EBV-associated cytokine storm with septic multi-organ dysfunction, and management followed a sepsis/hyperinflammation supportive pathway rather than HLH-specific chemo-immunotherapy.

Treatment included methylprednisolone (initially 10 mg/kg/day, tapered to 1 mg/kg/day), IVIG (2 g/kg),escalating broad-spectrum antibiotics (including vancomycin after MRSA was isolated from sputum on hospital day 10), valacyclovir, organ-supportive care, three sessions of continuous renal replacement therapy, and transfusion of washed red blood cells for persistent Coombs-positive hemolysis. The course was further complicated by ventilator-associated subcutaneous and mediastinal emphysema and, terminally, left-sided endophthalmitis, presumed to reflect hematogenous seeding from MRSA bacteremia.

### 2.4 Terminal course and outcome

On hospital day 13, the patient developed acute anisocoria, with a fixed and dilated right pupil (6 mm, absent light reflex). Emergent computed tomography of the head confirmed multifocal intraparenchymal hemorrhage predominantly involving the left frontal lobe, intraventricular hemorrhage, and midline shift to the right, consistent with cerebral herniation. Transcranial Doppler demonstrated bilaterally high-resistance flow patterns, consistent with markedly elevated intracranial pressure. Following discussion of the poor prognosis, the family elected to discontinue further intensive care and the patient was discharged; she died shortly after returning home.

## 3 Discussion

This case extends the phenotypic spectrum of *METTL23*-related disorder well beyond its originally described neurodevelopmental presentation (Table 1). Over three years of follow-up, the same patient whose intellectual disability phenotype we previously reported (6) developed recurrent autoimmune hemolytic anemia and, ultimately, a fatal Epstein–Barr virus-triggered hyperinflammatory episode. To our knowledge, this is the first report of either recurrent autoimmune cytopenia or a cytokine storm-like presentation in a patient with a biallelic *METTL23* variant.

**Table 1:** Evolution of the phenotypic spectrum associated with biallelic METTL23 variants.

System/Domain	Key reference(s)	Core finding	Relevance to the present case
Neurodevelopmental (original/core phenotype)	Reiff 2014 (1); Bernkopf 2014 (2); Almannai 2020 (3); Khan 2020 (4); Smaili 2020 (5); Zha 2024 (6) (this patient)	Global developmental delay, mild-to-moderate cognitive impairment, variable facial dysmorphism; corpus callosum thinning and delayed white matter myelination on neuroimaging; behavioral abnormalities (ADHD, aggression) and seizures in a subset	Baseline phenotype of the present patient; detailed neurodevelopmental and neuroimaging findings reported previously (6)
Ophthalmologic (mechanistic insight)	Pan 2022 (7); Scheetz 2024 (8)	Normal-tension glaucoma; METTL23 catalyzes histone H3R17 dimethylation, required for pS2 transcription, which negatively regulates NF-*κ*B–mediated TNF-α/IL-1β feedback	Provides a plausible molecular mechanism linking METTL23 loss-of-function to dysregulated proinflammatory signaling (see Discussion)
Cardiovascular/immune (single-case observation)	Maeda 2025 (9)	IVIG-resistant Kawasaki disease with giant coronary artery aneurysms in a boy with METTL23-related developmental delay	First independent suggestion of inflammatory/vasculitic susceptibility in a METTL23 variant carrier
Hematologic/systemic inflammatory (novel, this report)	This case	Recurrent autoimmune hemolytic anemia (3 episodes over 3 years); fatal EBV-triggered cytokine storm with septic multi-organ dysfunction, fulfilling several but not all HLH-2004 criteria	First report of recurrent autoimmune cytopenia and fatal hyperinflammation in a METTL23 variant carrier; extends the phenotypic spectrum beyond neurodevelopment

HLH, hemophagocytic lymphohistiocytosis; IVIG, intravenous immunoglobulin; TNF-α, tumor necrosis factor alpha; IL-1β, interleukin-1 beta; NF-κB, nuclear factor kappa B. This table summarizes the evolving phenotypic spectrum across the published literature on METTL23-related disorder rather than individual genotype-phenotype correlations, which have been detailed for the neurodevelopmental phenotype elsewhere (6).

Although several laboratory markers during the final admission overlapped with HLH-2004 criteria (hyperferritinemia, hypofibrinogenemia, elevated soluble IL-2 receptor) (10), fewer than five of the eight diagnostic criteria were objectively met (Figure 2). Splenomegaly and marrow hemophagocytosis were absent throughout the illness, and the transient, isolated thrombocytopenia observed on hospital day 5 did not constitute sustained bicytopenia; anemia could not be reliably scored as an HLH-related cytopenia given the patient's underlying chronic autoimmune hemolytic anemia. NK-cell percentage, rather than functional activity, was measured and could not be scored against the corresponding criterion. The presentation therefore fell short of a formal HLH diagnosis both clinically and numerically, and the patient was managed as EBV-associated cytokine storm with septic multi-organ dysfunction rather than with HLH-specific chemo-immunotherapy. We emphasize this distinction because the finding of a *METTL23* variant in a patient with a severe but sub-threshold hyperinflammatory episode is, in itself, informative — it suggests that the immune dysregulation observed need not rise to full HLH to be clinically and mechanistically relevant.

**Figure 2:**
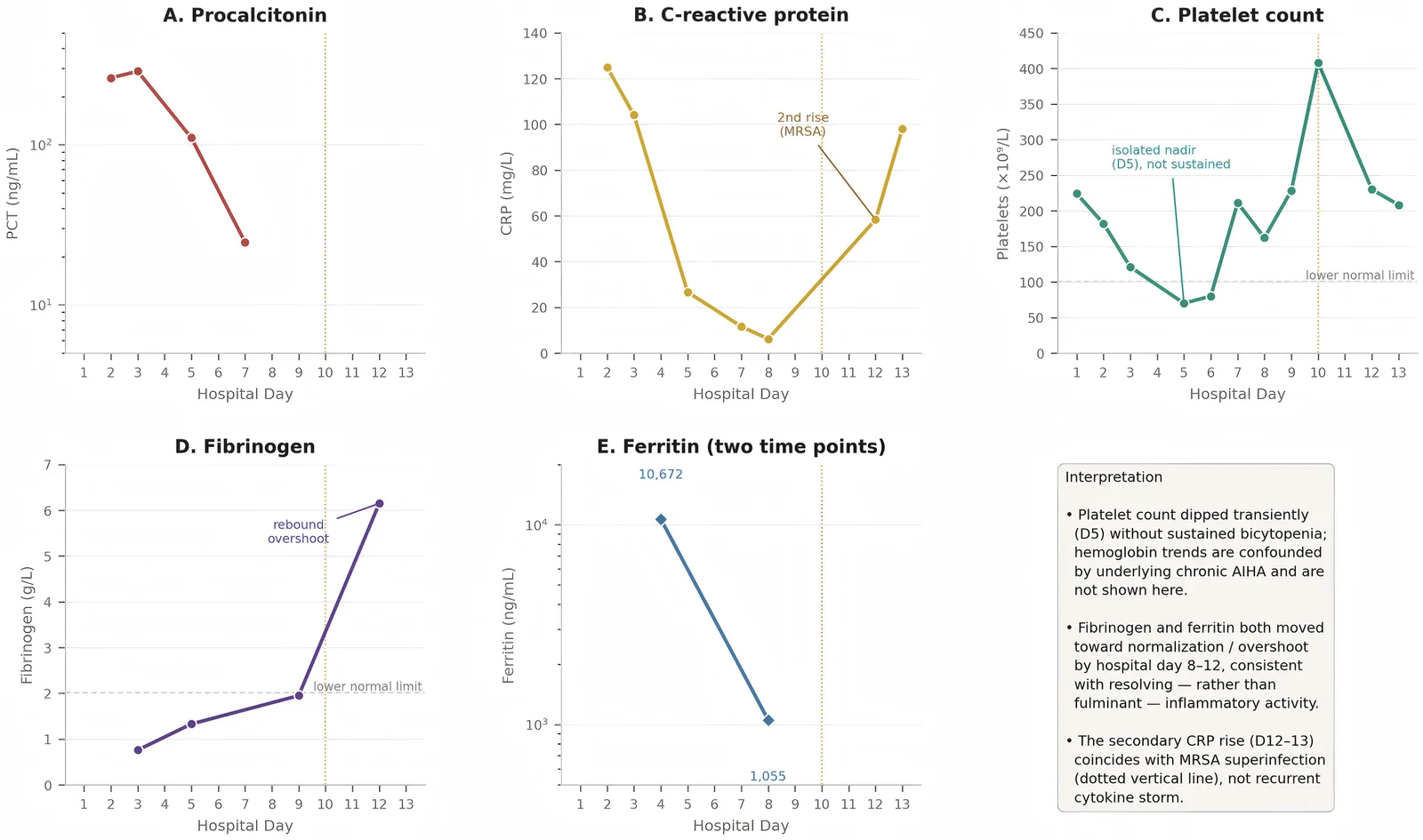
Dynamics of inflammatory and HLH-associated laboratory markers during the final hospitalization.

A plausible mechanistic link between METTL23 and dysregulated inflammation comes from Pan et al., who demonstrated that METTL23 catalyzes dimethylation of histone H3R17 and is required for transcription of *pS2*, which in turn negatively regulates NF-*κ*B–mediated TNF-α and IL-1β feedback in retinal ganglion cells (7), a finding subsequently discussed as an example of the broader contribution of histone methylation to tissue-specific and neurodegenerative disease mechanisms (11–13). If a comparable regulatory relationship exists in immune cells, loss of METTL23 function could be expected to attenuate this negative feedback loop, lowering the threshold at which an infectious trigger — EBV in the present case — precipitates excessive NF-*κ*B–driven cytokine production. This hypothesis remains speculative: it is extrapolated from a single tissue-specific mechanistic study in retinal tissue, and we did not perform functional studies (e.g., cytokine profiling, NF-*κ*B pathway activity, or NK-cell cytotoxicity assays) in this patient or her family to test it directly.

Further, albeit indirect, support for this hypothesis comes from the position of *METTL23* within the broader methyltransferase-like (METTL) gene family. The METTL family comprises over 30 seven-*β*-strand, S-adenosylmethionine-dependent methyltransferases in humans, which can be broadly divided into nucleic-acid-modifying members (e.g., *METTL3/METTL14*, which catalyze N6-methyladenosine modification of mRNA and have well-documented roles in NF-*κ*B-related inflammatory and immune signaling) and a phylogenetically distinct clade of protein lysine methyltransferases (14). *METTL23* belongs to the latter group, clustering most closely with *METTL21A–E* and *METTL22*, enzymes that predominantly target molecular chaperones and other stress-responsive proteins (e.g., HSP70, GroEL, and the DNA repair/RNA metabolism protein Kin17) (14). This family-level pattern suggests that *METTL23* operates within a functional module broadly involved in cellular stress and proteostasis regulation, distinct from — but potentially complementary to — the RNA-modifying METTL members already implicated in inflammatory signaling. While this evolutionary relationship does not establish a direct immune function for METTL23, it provides additional biological plausibility for the hypothesis that its loss of function could affect stress- and infection-responsive pathways, beyond the single mechanistic link described above.

Independent clinical support for a possible inflammatory phenotype in *METTL23*-related disorder comes from a case reported by Maeda et al., in which a boy with *METTL23*-related developmental delay developed intravenous immunoglobulin-resistant Kawasaki disease with giant coronary artery aneurysms — itself a cytokine-driven vasculitic syndrome of childhood (9). Considered together with the present case, these two independent observations, in unrelated patients from different countries, raise the possibility that *METTL23* loss-of-function may confer a broader susceptibility to dysregulated inflammatory responses upon immune triggers, rather than representing coincidental comorbidity. We also note that the patient's recurrent autoimmune hemolytic anemia — itself an antibody-mediated immune process — preceded the final hyperinflammatory episode by several years, raising the question of whether a shared underlying immune dysregulation, rather than two unrelated conditions, better explains the overall clinical course; this cannot be resolved with the data available.

This report has several limitations inherent to a single case. First, we cannot establish causality between the *METTL23* variant and the hyperinflammatory phenotype; population-level or functional data would be required to confirm this association, and it remains possible that the observations in this patient and in the Kawasaki disease case (9) reflect chance co-occurrence in a population where EBV infection and Kawasaki disease are themselves common. Second, no cytokine panel, NK-cell functional assay, or genetic testing for canonical HLH-associated genes (e.g., *PRF1, UNC13D, STX11, STXBP2*) (10) was performed during the final admission. Because the patient died and no blood samples, cells, or DNA were retained, these analyses cannot be performed retrospectively, nor can splicing (RNA) studies be performed to confirm the effect of the c.322 + 1del variant. Our mechanistic hypothesis therefore requires validation in patient-derived cells or animal models in future cases. Third, as a retrospective single-patient report, we cannot determine the generalizability of these findings to other patients with *METTL23*-related disorder.

Despite these limitations, this case suggests that patients with *METTL23*-related disorder may benefit from long-term multidisciplinary surveillance that extends beyond neurodevelopmental follow-up to include hematologic and immunologic monitoring, with a low threshold for evaluating hyperinflammatory features during intercurrent infections such as EBV. Further studies — including functional immune characterization of patients with *METTL23* variants and systematic collection of additional cases — are needed to determine whether the association proposed here reflects a genuine, generalizable feature of this rare disorder.

## 4 Conclusion

This case extends the phenotypic spectrum of *METTL23*-related disorder beyond its originally described neurodevelopmental presentation to include recurrent autoimmune cytopenia and, ultimately, a fatal Epstein–Barr virus-triggered cytokine storm with septic multi-organ dysfunction — a presentation that overlapped with, but did not formally meet, HLH-2004 diagnostic criteria. Considered alongside a prior independent report of Kawasaki disease in another child with a *METTL23* variant, the known role of METTL23 in restraining NF-*κ*B–mediated proinflammatory signaling, and its phylogenetic position within a chaperone-associated clade of protein methyltransferases, this case raises the hypothesis that *METTL23* loss-of-function may confer susceptibility to dysregulated inflammatory responses beyond the nervous system. This hypothesis remains to be confirmed by functional studies and additional cases, but in the interim, long-term multidisciplinary follow-up — extending beyond neurodevelopmental surveillance to include vigilance for infection-triggered hyperinflammation — may be warranted in patients with *METTL23*-related disorder.

## Data Availability

The original contributions presented in the study are included in the article. The raw sequencing data are not publicly available due to patient privacy and consent restrictions; further inquiries can be directed to the corresponding author.
